# Vitamin D Metabolic Profiles in Premenopausal Women Wearing Niqab and Hijab in Sunny Basrah

**DOI:** 10.7759/cureus.14909

**Published:** 2021-05-08

**Authors:** Samih A Odhaib, Nassar T Alibrahim, Ibraheem A Zaboon, Abbas A Mansour

**Affiliations:** 1 Adult Endocrinology, Faiha Specialized Diabetes, Endocrine and Metabolism Center (FDEMC) College of Medicine, University of Basrah, Basrah, IRQ

**Keywords:** basrah, calcium, hijab, hypovitaminosis, niqab, parathormone, vitamin d deficiency, dress style

## Abstract

Background

Conservative clothing like niqab and hijab may affect the wearer’s vitamin D metabolic parameters even in predominantly sunny areas of the world (i.e., areas with adequate sunlight exposure throughout the year). Our objective was to evaluate the effect of wearing the niqab or hijab on different vitamin D3 metabolic parameters in a sample of premenopausal women from Basrah.

Methodology

This was a cross-sectional observational study on premenopausal women who wore a niqab (n = 64), with a comparable age-matched group of women who wore the hijab (n = 60). Biochemical evaluation of the vitamin D3 metabolic profile involved 25-hydroxycholecalciferol (25-OH)-vitamin D, corrected serum calcium, parathyroid hormone (PTH), phosphorus, and alkaline phosphatase estimation. Statistical comparison of these parameters was made using the independent sample t-test and Mann-Whitney-U test.

Results

The two groups of women were age- and weight-matched, with a median age of 39 and median body mass index (BMI) of 31.8 kg/m^2^. Overall, age, marital status, and BMI of women in both groups had no significant relationship with the vitamin D3 metabolic parameters (low 25-OH-vitamin D, low corrected calcium, and high PTH). The subgroup analysis for women wearing the niqab showed the same results.

Conclusions

Wearing niqab or hijab in premenopausal women was not associated with any significant statistical relationship or difference in vitamin D3 metabolic parameters. Conservative dress styles like niqab and hijab are practical barriers to sun exposure and contribute to suboptimal vitamin D levels, affecting subsequent metabolic pathways. Healthcare professionals should advise women who wear niqab or hijab to increase their vitamin D3 levels through culturally appropriate alternative mechanisms.

## Introduction

According to religious and cultural norms, conservative Muslim women’s dress styles range from dark, solid-colored, whole-body covering, including the hands and face (niqab), to more common styles that exclude the hands and face (hijab), which can be dark or colorful. Women typically wear the niqab or hijab outdoors but not inside their homes [1].

Women wearing a hijab or niqab have insufficient skin surface area for sun exposure. Consequently, they experience less vitamin D3 synthesis compared to women wearing a western-style dress [2-5] because hijab and niqab dress styles attenuate the penetration of ultraviolet B (UVB) light needed for vitamin D3 synthesis [3,5].

The prevalence of hypovitaminosis D is 1.6 to 1.7 times higher for women wearing a hijab and 1.9 to 2.7 times higher for women wearing a niqab than women in western dress styles [1,6]. Hypovitaminosis D carries a greater risk of osteoporosis [7].

The main predictors for hypovitaminosis D include increasing age, female gender, multiparity, high body mass index (BMI), low physical activity levels, low intake of calcium or vitamin D supplements, concealed clothing, low sun exposure duration (low UVB doses), winter season, low socioeconomic status, urban residence, skin pigmentation, and chronic comorbidities [8-11].

We evaluated the effect of wearing niqab and hijab on the vitamin D status in a sample of Iraqi women from Basrah, Iraq.

The abstract of this article was submitted as an e-poster in the American Association of Clinical Endocrinology Gulf Chapter Virtual Annual Meeting (AACE GC) conducted in November 2020. The abstract was also submitted as an e-poster at the ENDO2021 of the Endocrine Society conducted in March 2021.

## Materials and methods

We conducted a cross-sectional observational study on premenopausal women who wore the niqab and attended Faiha Specialized Diabetes, Endocrine and Metabolism Center (FDEMC) in Basrah, Iraq, in 2019, for consultation for menstrual irregularities. Women with the niqab who had continued wearing the niqab in the last three years were enrolled initially in the study (n = 64).

The study excluded women with a history of chronic comorbidities (e.g., diabetes mellitus, thyroid diseases, adrenal diseases, celiac disease, inflammatory bowel disease, malignancies, and lactose intolerance), pregnancy or lactation in the last three years, current or past history of regular use of hormonal medications (e.g., steroids, antipsychotics, heparin, anti-resorptives, and contraceptive medications), history of recent fractures, history of any skeletal disability or wheel-chair use for any indication, history of any recent operative intervention, and those with regular outdoor work. All the enrolled women were unemployed or did not work away from home for at least three years, with no regular outdoor work.

We evaluated the enrolled women using thorough history-taking, physical examinations, and biochemical investigations. We collected demographic and general characteristics (e.g., age, weight, BMI, and marital status) and assessed 25-hydroxycholecalciferol (25-OH)-vitamin D, corrected serum calcium, phosphorus, and parathyroid hormone (PTH). We also evaluated thyroid hormone levels, alkaline phosphatase (ALP), lactate dehydrogenase, and creatinine phosphokinase for enrollment and exclusion purposes.

We enrolled 64 women who wore the niqab (i.e., the niqab group). Another age-matched 64 women who wore the hijab were selected as a comparison group (i.e., the hijab group or control group), who presented at the same time and met the criteria for patient selection. The randomization in the age-matched hijab group was done on a 1:1 basis. Four women in the hijab group were considered as defaulters and were excluded from the study.

The niqab in Iraq is a black outfit covering the whole body, even the eyes, while the hijab can feature multiple colors and covers the whole body except the face, hands, and (sometimes) feet. Women who wear hijab have 8% skin exposure, while skin exposure is null for women who wear the niqab [12].

Laboratory investigations

Study participants underwent an early morning venous sample following a minimum eight-hour overnight fasting. A 10-mL sample of venous blood was collected between 8:00 AM and 10:00 AM and kept in a gel tube, then centrifuged at 4,100 xg in a NÜVE-NF 800 centrifuge (NÜVE, Ankara, Turkey). The 25-OH-vitamin D and PTH were assessed using the electrochemiluminescence technology of a Cobas e411 Analyzer (Roche, Germany). The reference values for 25-OH-vitamin D were <50 nmol/L (deficiency), 52 to 70 nmol/L (insufficient), and >70 to 150 nmol/L (sufficient). The reference range of PTH was 1.5 to 6.5 pmol/L. Serum calcium, albumin, phosphorus, and ALP levels were measured using a Cobas INTEGRA 400 plus analyzer (Roche, Germany). The serum reference ranges were as follows: calcium, 2.1 to 2.6 mmol/L; albumin, 35 to 50 g/L; ALP, 0.68 to 2.55 µkat/L; and phosphorus, 0.99 to 2.02 mmol/L.

Ethical approval and consent

All women enrolled in the study provided informed verbal and written consent. The study was conducted in accordance with the ethical standards of the FDEMC research committee, from which the ethical approval was obtained, and followed the 1964 Declaration of Helsinki and its later amendments or comparable ethical standards.

Statistical analysis

We analyzed the data using IBM SPSS Statistics for Windows, version 26.0. (IBM Corp., Armonk, NY). The study used the mean ± standard deviation or frequency (%) for data expression. For continuous variables, we used the independent t-test and the Mann-Whitney U-test. Scatter plots were used to demonstrate different relationships between 25-OH-vitamin D and other variables. A two-tailed p ≤ 0.05 was considered statistically significant at a 95% confidence interval.

## Results

The vast majority of women who attended FDEMC in 2019 wore the hijab, and a minority wore the niqab. No women wore a western-style dress.

Table 1 presents demographic information for both groups. The median age was 39 years (age range: 20-50 years), and the mean age and distribution of women across age groups were similar and did not differ significantly. The age of women in both groups had no significant relationship with vitamin D3 metabolic parameters (low 25-OH-vitamin D, high PTH, and low corrected calcium levels).

**Table 1:** General characteristics of the women in the niqab and hijab groups. ^a^t-test; ^b^Mann-Whitney U-test ALP: alkaline phosphatase; BMI: body mass index; PTH: parathyroid hormone; SD: standard deviation

Variables		Niqab (n = 64)	Hijab (n = 60)	P-Value
Age	Mean ± SD years	39.0 ± 8.9	38.8 ± 9.0	0.916^a^
	≤39 years n (%)	33 (51.6)	30 (50.0)	0.862
	>39 years n (%)	31 (48.4)	30 (50.0)	
Marital status	Married n (%)	56 (87.5)	43 (71.7)	0.028
	Unmarried n (%)	8 (12.5)	17 (28.3)	
BMI kg/m^2^	Mean ± SD	31.8 ± 6.7	31.8 ± 6.0	0.998^a^
	Obese (BMI ≥ 30) n (%)	34 (53.1)	36 (60.0)	0.440
	Non-obese (BMI < 30) n (%)	30 (46.9)	24 (40.0)	
25-OH-vitamin D nmol/L	Mean ± SD	28.70 ± 24.46	37.19 ± 26.46	0.067^b^
	Low D3 n (%)	57 (89.1)	47 (78.3)	0.105
Corrected calcium mmol/L	Mean ± SD	2.2 ± 0.13	2.2 ± 0.20	0.649^a^
	Low corrected calcium n (%)	16 (25.0)	13 (21.7)	0.661
PTH pmol/L	Mean ± SD	5.64 ± 2.60	5.80 ± 2.96	0.747^a^
	High PTH n (%)	23 (35.9)	20 (33.3)	0.761
Phosphorus mmol/L	Mean ± SD	1.51 ± 0.26	1.55 ± 0.43	0.641^b^
ALP µkat/L	Mean ± SD	1.45 ± 0.56	1.41 ± 0.50	0.550^b^

There was a significant difference in marital status between the niqab and hijab group; there were more married women in both groups and more in the niqab group than the hijab group. The marital status of women in both groups had no significant difference in the frequency of low 25-OH-vitamin D. Unmarried women who wore the niqab had a 10-fold risk of having high PTH compared to women who wear hijab.

Tables 2, 3 elucidate the effect of different factors (e.g., age, marital status, and weight) on vitamin D3 metabolic parameters as categorical variables and continuous variables, respectively. BMI did not affect the distribution of cases between groups; the sample was nearly weight-matched. The only significant difference was seen in non-obese (lean) women in the niqab group who have an odds ratio of 7.0. Non-obese niqab wearers were seven times more prone to have hypovitaminosis D3 than non-obese hijab wearers. We found no significant difference between the 25-OH-vitamin D levels in the two groups. We noticed more women with hypovitaminosis D3 and lower mean 25-OH-vitamin D levels in the niqab group than women in the hijab group. This was evident in the lack of significant difference in the mean PTH and the number of women with niqab who had high PTH levels (i.e., 23 women with niqab had high PTH versus 20 women with hijab).

**Table 2:** Factors affecting the frequency of some vitamin D3 metabolic parameters between women in the niqab and hijab groups. ^a^The obesity range includes BMI ≥ 30 kg/m^2^, and non-obesity includes BMI < 30 kg/m^2^ BMI: body mass index; CI: confidence interval; OR: odds ratio; PTH: parathyroid hormone

Parameters of vitamin D3 metabolism		Niqab group (n = 64)	Hijab group (n = 60)	OR	95% CI	P-Value
Low 25-OH-vitamin D						
Age years n (%)	≤39	30 (90.9)	25 (83.3)	2.00	0.435-9.205	0.367
	>39	27 (87.1)	22 (73.3)	2.46	0.652-9.241	0.176
Marital status n (%)	Married	49 (87.5)	32 (74.4)	2.41	0.84-6.856	0.094
	Unmarried	8 (100.0)	15 (88.2)	1.13	0.953-1.348	0.312
BMI n (%)^ a^	Obese	29 (85.3)	31 (86.1)	0.94	0.245-3.569	0.922
	Non-obese	28 (93.3)	16 (66.7)	7.00	1.322-37.066	0.012
Low corrected calcium						
Age years n (%)	≤39	9 (27.3)	7 (23.3)	1.32	0.394-3.858	0.720
	>39	7 (22.6)	6 (20.0)	1.17	0.342-3.985	0.806
Marital status n (%)	Married	12 (21.4)	8 (18.6)	1.19	0.440-3.239	0.729
	Unmarried	4 (50.0)	5 (29.4)	2.40	0.423-13.601	0.317
BMI n (%)^ a^	Obese	7 (20.6)	7 (19.4)	1.07	0.333-3.466	0.905
	Non-obese	9 (30.0)	6 (25.0)	1.29	0.384-4.310	0.684
High PTH						
Age years n (%)	≤39	15 (45.5)	11 (36.7)	1.44	0.524-3.954	0.479
	>39	8 (25.8)	9 (30.0)	0.81	0.265-2.490	0.715
Marital status n (%)	Married	16 (28.6)	13 (30.2)	0.923	0.386-2.207	0.857
	Unmarried	7 (87.5)	7 (41.2)	10.00	0.995-100.462	0.030
BMI n (%) ^a^	Obese	14 (41.2)	11 (30.6)	1.59	0.594-4.258	0.354
	Non-obese	9 (30.0)	9 (37.5)	0.71	0.229-2.227	0.561

**Table 3:** Factors affecting the mean of some parameters of vitamin D3 metabolism in women in the niqab and hijab groups. ^a^Mann-Whitney U-test; ^b^t-test; ^c^The obesity range included BMI ≥ 30 kg/m^2^, and non-obesity range included BMI < 30 kg/m^2^ BMI: body mass index; PTH: parathyroid hormone; SD: standard deviation

Metabolic parameters of vitamin D3			Niqab group Mean ± SD	Hijab group Mean ± SD	P-Value
Low 25-OH-vitamin D nmol/L	Age	≤39 years	26.21 ± 16.22	34.45 ± 26.46	0.386^a^
		>39 years	31.70 ± 30.95	40.19 ± 26.71	0.104^a^
	Marital status	Married	27.21 ± 25.46	41.43 ± 27.96	0.003^a^
		Unmarried	39.44 ± 8.24	26.46 ± 18.97	0.023^a^
	BMI^c^	Obese	30.45 ± 5.24	33.20 ± 3.74	0.202^a^
		Non-obese	26.96 ± 13.98	43.18 ± 31.70	0.015^b^
Low corrected calcium mmol/L	Age	≤39 years	2.18 ± 0.10	2.20 ± 0.23	0.405^a^
		>39 years	2.23 ± 0.13	2.23 ± 0.15	0.729^a^
	Marital status	Married	2.20 ± 0.10	2.23 ± 0.15	0.527^a^
		Unmarried	2.20 ± 0.18	2.6 ± 0.25	0.793^a^
	BMI^c^	Obese	2.20 ± 0.13	2.23 ± 0.18	0.687^b^
		Non-obese	2.20 ± 0.10	2.2 ± 0.20	0.774^a^
High PTH pmol/L	Age	≤39 years	6.41 ± 2.81	5.69 ± 3.00	0.325^b^
		>39 years	4.82 ± 2.10	5.91 ± 2.95	0.099^b^
	Marital status	Married	5.38 ± 2.46	5.72 ± 2.74	0.587^a^
		Unmarried	7.46 ± 2.99	6.01 ± 3.53	0.308^a^
	BMI^c^	Obese	6.06 ± 2.90	5.61 ± 2.67	0.496^b^
		Non-obese	5.16 ± 2.15	6.09 ± 3.38	0.224^b^

The mean level and the total number of women with low corrected calcium, mean phosphorus, and mean ALP was not significantly different.

Table 4 presents the effect of age, marital status, and weight on various vitamin D metabolic markers in the niqab group only, and Table 5 lists categorical and continuous variables. There were no significant differences in these parameters in women of different ages and weights. Marital status did not affect the number of women wearing niqab with low 25-OH-vitamin D and corrected calcium. Unmarried women wearing niqab had significantly higher 25-OH-vitamin D levels than married women (39.44 nmol/L ± 8.24 nmol/L versus 27.21 nmol/L ± 25.46 nmol/L), yet both married and unmarried women had low 25-OH-vitamin D levels. Unmarried women also had significantly higher PTH levels than married women wearing a niqab (7.46 pmol/L ± 2.99 pmol/L versus 5.38 pmol/L ± 2.46 pmol/L. Age did not affect 25-OH-vitamin D or corrected calcium; its significant effect was only on the PTH levels (i.e., the lower the age, the higher the PTH level). The PTH level of women aged 39 or younger was more than women older than 39 (6.41 pmol/L ± 2.81 pmol/L versus 4.82 pmol/L ± 2.10 pmol/L).

**Table 4:** Factors affecting the frequency of vitamin D3 metabolic parameters in women with niqab only. BMI: body mass index; CI: confidence interval; OR: odds ratio; PTH: parathyroid hormone

Variables		Low 25-OH-vitamin D	Low corrected calcium	High PTH
Age	≤39 years n (%)	30 (90.9)	9 (27.3)	15 (45.5)
	>39 years n (%)	27 (87.1)	7 (22.6)	8 (25.8)
	OR	1.481	1.286	2.396
	95% CI	0.304-7.226	0.412-4.013	0.833-6.893
	P-Value	0.625	0.665	0.102
Marital status	Married n (%)	49 (87.5)	12 (21.4)	16 (28.6)
	Unmarried n (%)	8 (100.0)	4 (50.0)	7 (87.5)
	OR	0.875	0.273	0.057
	95% CI	0.793-0.966	0.059-1.254	0.006-0.502
	P-Value	0.289	0.081	0.001
BMI	Obese (BMI ≥ 30 kg/m^2^) n (%)	29 (85.3)	7 (20.6)	14 (41.2)
	Non-obese (BMI < 30 kg/m^2^) n (%)	28 (93.3)	9 (30.0)	9 (30.0)
	OR	0.414	0.605	1.633
	95% CI	0.074-2.314	0.193-1.893	0.579-4.609
	P-Value	0.304	0.386	0.352

**Table 5:** Factors affecting the mean of parameters of vitamin D3 metabolism in women wearing niqab only. All variables are expressed as (mean ± standard deviation). ^a^SI units of 25-OH-vitamin D, corrected calcium, and PTH are nmol/L, mmol/L, and pmol/L, respectively; ^b^Mann-Whitney U-test; ^c^t-test BMI: body mass index; 25-OH: 25-hydroxycholecalciferol; PTH: parathyroid hormone

Parameters		25-OH-vitamin D^a^	Corrected calcium^a^	PTH^a^
Age	≤39	26.21 ± 16.22	2.18 ± .10	6.41 ± 2.81
	>39	31.70 ± 30.95	2.23 ± 0.13	4.82 ± 2.10
	P-Value	0.696^b^	0.228^c^	0.013^c^
Marital status	Married	27.21 ± 25.46	2.20 ± 0.10	5.38 ± 2.46
	Unmarried	39.44 ± 8.24	2.20 ± 0.18	7.46 ± 2.99
	P-Value	0.012^b^	0.920^c^	0.022^c^
BMI	Obese	30.45 ± 5.24	2.20 ± 0.13	6.06 ± 2.90
	Non-obese	26.96 ± 2.50	2.20 ± 0.10	5.16 ± 2.15
	P-Value	0.787^b^	0.458^c^	0.169^c^

**Figure 1:**
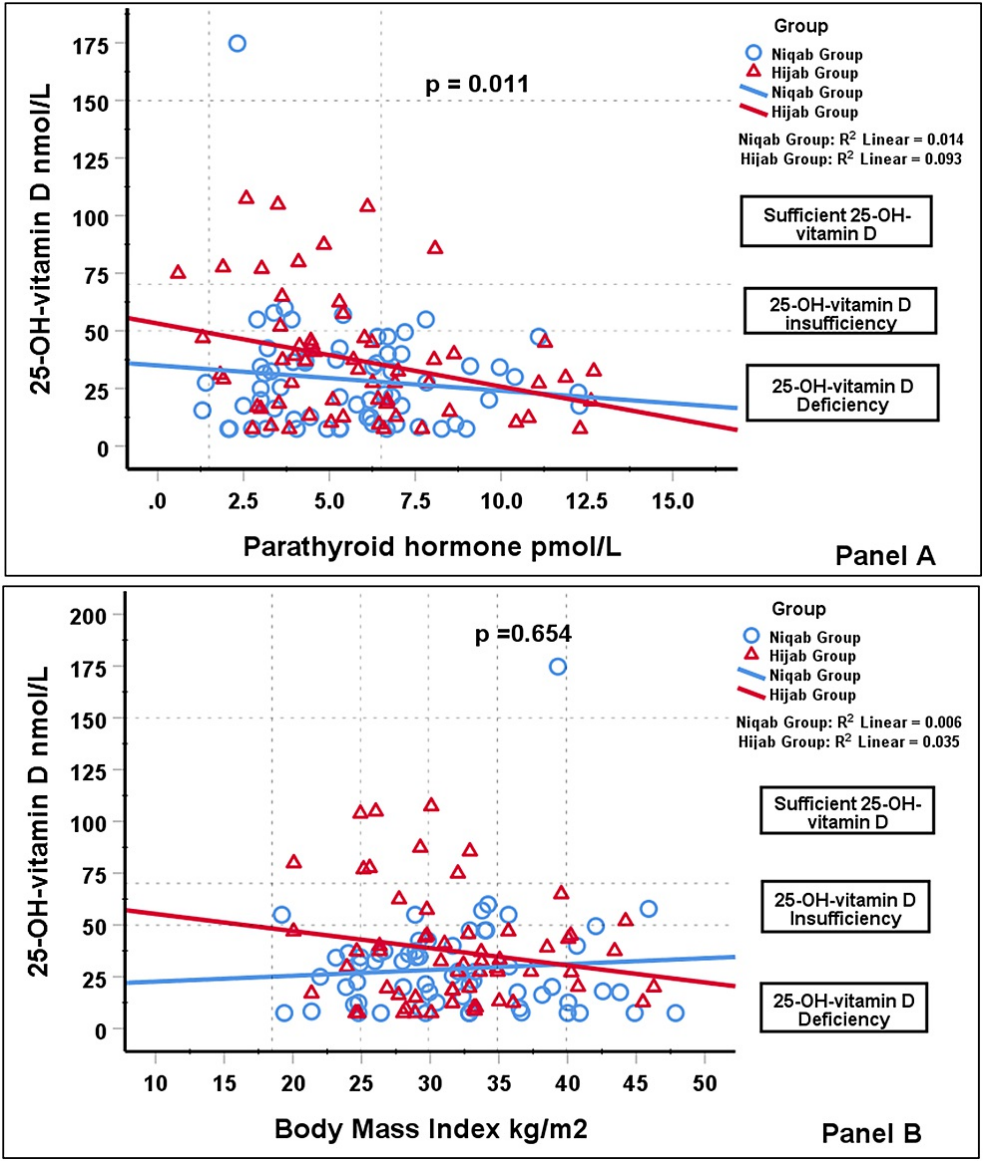
Panel A demonstrates the significant inverse linear relationship between the 25-OH-vitamin D with the different PTH levels for women wearing niqab and hijab. Panel B demonstrates the non-significant linear relationship between 25-OH-vitamin D at different BMI levels for women wearing niqab and hijab. The relationship was inverse in women with hijab, while it increased as the BMI increased, although no specific pattern can be deduced in women wearing niqab. Most women had either a deficient or insufficient level of 25-OH-vitamin D. There was one extreme 25-OH-vitamin D value for a woman with a niqab above the upper limit of the reference range. The dotted lines in the figure represent the different limits of the reference ranges of 25-OH-vitamin D, PTH, and BMI. BMI: body mass index; PTH: parathyroid hormone; 25-OH-vitamin D: 25-hydroxycholecalciferol vitamin D

Figure 1A presents the relationship between the 25-OH-vitamin D level and the PTH in both groups of women. We found a significant inverse relationship between the two variables in both groups of women. The reduction in the level of 25-OH-vitamin D was accompanied by an increase in the level of PTH in both groups in a similar pattern (i.e., D3 = 34.97-1.1 × PTH in women with niqab, and D3 = 53.15-2.74 × PTH in women with hijab). Figure 1B illustrates the relationship between 25-OH-vitamin D level and BMI in both groups of women. The distribution was unclear and lacked any significance in both groups. The 25-OH-vitamin D increased with increasing BMI in women wearing niqab (D3 = 19.82 + 0.28 × BMI), while it decreased as the BMI increased in women wearing hijab (63.59 - 0.83 × BMI).

## Discussion

Although Basrah, with its tropical latitude (30.5°N, 47.8°E), enjoys a sunny climate throughout the year [13], direct exposure to sunlight by the local population is limited due to high daytime temperatures, which may reach up to 50°C in summer. Most individuals stay indoors during the day with minimal sunlight exposure associated with low vitamin D levels among women.

The low sunlight exposure is a common problem in many Middle East countries with abundant sunlight. Previous reports attributed hypovitaminosis D to limited sunlight exposure, low vitamin D intake, indoor work environment, concealed dress styles that cover most of the body (including the face, head, and arms), parity, and the prevailing social norms that the women are home-bound and leave only when necessary, often with permission from the husband or guardian [1,3,14-16]. Subnormal serum 25-OH-vitamin D levels were found in women wearing coverings and long clothing that might have interfered with penetration of UVB radiation into the skin even when exposed to sunlight which affects the cutaneous synthesis of vitamin D3 [17]. Other studies suggested that the dress style was not the sole marker for hypovitaminosis D [3,18,19].

Approximately 89% of women wearing a niqab in our cohort (n = 57) had hypovitaminosis D3 compared to 78% of women wearing hijab (n = 47). The relationship between the level of 25-OH-vitamin D and the dress style was not significant. Hypovitaminosis D in women wearing the niqab or hijab was consistent with previous studies [1,3,6,16,20]. Batieha et al. found that the relation between hypovitaminosis D and exposed skin area was close to scientific significance. However, women wearing hijab and niqab did not differ significantly in their mean serum 25-OH-vitamin D levels [3].

There was no significant difference in PTH levels for either dress style, and we found no significant difference in the number of women with high PTH by dress style. All changes encountered initially are insignificant, regardless of dress style. Still, more women with corresponding changes in 25-OH-vitamin D and PTH in the niqab group were encountered. Similar data for the effect of low 25-OH-vitamin D and PTH were reported from many Middle Eastern countries with mean serum 25-OH-vitamin D lower in the niqab group compared to the hijab group, in whom indoor employment may play a role. Many studies adopted the lowest conservative ranges for vitamin D3, with a higher set point or physiologic adaptation of vitamin D, which may upregulate PTH [1,3,21,22]. Other studies also reported a non-significant relationship between vitamin D and PTH [7,11,18,23,24].

The significant inverse relationship between 25-OH-vitamin D and PTH in Figure 1A indicated hypovitaminosis D in both groups. The result was similar to those reported in two Jordanian studies [3,4]. The cut-off values of 25-OH-vitamin D, which mark the deficiency and insufficiency status, overlap [25]. It had been proposed that 30 to 125 nmol/L of 25-OH-vitamin D is required to maintain normal PTH levels, with most studies reporting values of approximately 75 to 80 nmol/L such as Al-Mogbel et al. [21], Grant et al. [25], and Wat et al. [26]. The biochemical assays to estimate 25-OH-vitamin D and PTH affect the correlation between the two variables and other variables, including latitude, diet, race, age, and gender [27].

Although 25-OH-vitamin D levels were markedly reduced, PTH levels were not increased in most women, similar to findings reported by Haarburger et al. They suggested that 25-OH-vitamin D measurements should be requested when hypovitaminosis D is clinically suspected, irrespective of calcium and PTH results. The subnormal 25-OH-vitamin D levels were not always related to hypocalcemia or a rise in PTH levels [24]. The elevated PTH in the presence of normocalcemia might reflect a subclinical or mildly clinical hypovitaminosis D [5,18,24].

Hypocalcemia stimulates PTH secretion, which activates vitamin D synthesis. Vitamin D and PTH production enhance renal calcium reabsorption and calcium mobilization by bone resorption. However, hypercalcemia promotes a reduction in PTH secretion and decreases vitamin D synthesis and calcium mobilization. Hypercalcemia also stimulates parafollicular cells in the thyroid to secrete calcitonin, inhibit calcium mobilization from the bone, stimulate the excretion of calcium and phosphorous, and maintain calcium within normal levels [28].

We found a 10-fold risk for unmarried women wearing niqab to have high PTH than unmarried women wearing hijab. This observation was limited to seven women from each group. Although a statistical significance was achieved, we could not confirm it on a larger scale during our study, especially given the odds ratio of unmarried women with low 25-OH-vitamin D levels, which is the main contributing factor for high PTH. Batieha et al. concluded that married women were significantly less likely to have lower vitamin D levels than unmarried women, wearing any dress style (niqab, hijab, or western style) based on their higher sample size (3,624 married women versus 644 unmarried women) [3]. Marital status was not the sole determinant of vitamin D status.

Women’s age did not affect PTH level. Batieha et al. concluded that older women had significantly higher PTH than men; however, they did not evaluate PTH in subgroups of women with different dress styles [3].

During the initial evaluation, the groups were weight-matched, with no significant difference between both groups (Table 1). However, during subgroup analysis, the non-obese had significantly more risk (i.e., seven times) of having hypovitaminosis D, which is more evident in lean women with niqab than women in hijab (26.96 ± 13.98 nmol/L versus 43.18 ± 31.70 nmol/L, respectively). Overall, 84% of women had hypovitaminosis D3 (57 women with niqab, 47 women with hijab). Both groups had mean vitamin D levels that fell within the range of severe vitamin D deficiency. Figure 1B showed the pattern of decreasing 25-OH-vitamin D levels as the BMI increased in the hijab group, while for the niqab group, the pattern was different.

Other studies have compared the effect of weight or BMI on vitamin D3 and confirmed that the higher the BMI, the lower the vitamin D3 [4,6,14,29,30]. Still, these studies had higher ranges of vitamin D3 levels above the reference levels. Women wearing the niqab are less likely to participate in outdoor activities than those in the other studies.

The mean BMI in both groups was in the obese range. Abundant adipose tissue limits the bioavailability of 25-OH-vitamin D [14]. Therefore, the association between hypovitaminosis D and obesity remains inconclusive. The results indicate the presence of other contributory factors for hypovitaminosis D in lean women from both groups with a more complex relationship than vitamin D bioavailability and uptake.

Despite low 25-OH-vitamin D levels among the participants, our study showed that corrected serum calcium, phosphorus, and ALP levels were within the normal range in approximately 77% of women (n = 95) for serum calcium and all enrolled women for serum phosphorus and ALP. This finding might be attributed to compensatory high PTH levels.

We found no significant difference between the two groups in the mean or total incidence of low corrected calcium, phosphorus, and ALP, which are indicators of bone turnover. Although most of the cohort from both groups had low 25-OH-vitamin D levels, 25% of the niqab group (n = 16) had low corrected calcium compared to approximately 22% of the hijab group (n = 13), and there was no significant difference between the groups.

The mean corrected calcium in both groups was within the reference ranges with no significant relationship to the dress style, similar to studies reported in neighboring countries [14,18]. Other studies showed lower serum calcium levels associated with dress style [2,23], with a significant positive correlation between 25-OH-vitamin D and ionized calcium [20]. In most cases, the finding of normal serum biochemical parameters was interesting and alarming, as many osteomalacia and osteoporosis cases could be missed if serum 25-OH-vitamin D were not measured.

We chose age-matched groups of premenopausal women to overcome the possible physiological anabolic estrogen action on bones in both groups regardless of the dress style for this study. However, the study had many limitations. The small size may not represent the burden of hypovitaminosis D in these women, which was influenced by the total number of FDEMC attendees who wore the niqab, which was small. We did not have an accurate tool to measure daily calcium and vitamin D intake. The seasonal variation in sun exposure behavior was not evaluated. The effect on bone density and turnover markers was not tested due to its unnecessary cost, and there were no relevant indications. We did not have any reliable data about social norms or the women’s actual outdoor activities concerning the time spent indoors or outdoors. We did not study the social barriers that influence women to be home-bound most of the time. Despite these limitations, the seemingly homogeneous population in the present study was well documented in demographic and biochemical measurements.

## Conclusions

Clothing is an effective blocker to sun exposure and thus contributes to vitamin D synthesis and status. Given the low comparative exposed skin areas, wearing the niqab or hijab was not associated with significant differences in vitamin D3 metabolic parameters. The majority of study participants in both groups had hypovitaminosis D3 at presentation, albeit asymptomatic. Young unmarried women wearing niqab had significantly higher PTH levels in comparison to unmarried women wearing hijab. These women also showed significantly higher 25-OH-vitamin D levels, although it was deficient compared to married women wearing niqab or hijab. Healthcare providers should advise these women of precautionary measures to increase their vitamin D3 levels through culturally and religiously appropriate alternative mechanisms.
